# Supplementary material for: Contribution of leukocyte telomere length to cardiovascular disease onset from genome-wide cross-trait analysis
Source: Nat Commun. 2025 Sep 30;16:8677. doi: 10.1038/s41467-025-63707-5 (PMC12485159; doi:10.1038/s41467-025-63707-5)
Supplement: Supplementary file 1 — Supplementary Information [file 41467_2025_63707_MOESM1_ESM.pdf]

Contribution of leukocyte telomere length to cardiovascular disease onset from genome-wide cross-trait analysis

Jun Qiao[1,2,3#], Qian Wang[4#], Yuhui Zhao[5#], Minjing Chang[6#], Shuo Sun[7#], Pengwei Zhang[1,2,3], Kaixin Yao[8], Miaoran Chen[8], Leilei Zheng[9], Xiaolong Xing[5], Liuyang Cai[1,2,3], Anil G. Jegga[10,11,12], Lei Jiang[7✉], Siim Pauklin[13✉], Rongjun Zou[14,15,16✉], Yining Yang[17,18✉], Yuliang Feng[1,2,3✉]

[1] Department of Geriatrics, Shenzhen People's Hospital (The First Affiliated Hospital of Southern University of Science and Technology), Shenzhen, China.

[2] Department of Pharmacology, SUSTech Homeostatic Medicine Institute, School of Medicine, Southern University of Science and Technology, Shenzhen, China.

[3] Joint Laboratory of Guangdong-Hong Kong Universities for Vascular Homeostasis and Diseases, Shenzhen, China.

[4] Department of Nephrology, The Fifth Clinical Medical College of Shanxi Medical University, Taiyuan, China.

[5] Department of Rheumatology, Shanxi Key Laboratory of Immunomicroecology, Second Hospital of Shanxi Medical University, Taiyuan, China.

[6] School of Public Health and Emergency Management, School of Medicine, Southern University of Science and Technology, Shenzhen, China.

[7] Department of Cardiology, Guangdong Cardiovascular Institute, Guangdong Provincial People's Hospital (Guangdong Academy of Medical Sciences), Southern Medical University, Guangzhou, China.

[8] Department of Nephrology, Shanxi Kidney Disease Institute, Second Hospital of Shanxi Medical University, Taiyuan, China.

[9] Department of Otolaryngology-Head and Neck Surgery, Second Hospital of Shanxi Medical University, Taiyuan, China.

[10] Department of Pediatrics, University of Cincinnati College of Medicine, Cincinnati, USA.

[11] Department of Computer Science, University of Cincinnati College of Engineering, Cincinnati, USA.

[12] Department of Internal Medicine, College of Medicine, University of Cincinnati, Cincinnati,

USA.
[13] Botnar Research Centre, Nuffield Department of Orthopaedics, Rheumatology and Musculoskeletal Sciences, University of Oxford, Headington, Oxford, UK.
[14] State Key Laboratory of Traditional Chinese Medicine Syndrome, Guangdong Provincial Hospital of Chinese Medicine, Guangdong, China.
[15] State Key Laboratory of Dampness Syndrome of Chinese Medicine, Guangzhou, China.
[16] Guangdong Provincial Key Laboratory of TCM Emergency Research, Guangzhou, China.
[17] Department of Cardiology, People's Hospital of Xinjiang Uygur Autonomous Region, Urumqi, China.
[18] Xinjiang Key Laboratory of Cardiovascular Homeostasis and Regeneration Research, Urumqi, China.
[#]These authors contributed equally to this work.

Supplementary Figures and Notes **Page**

Supplementary Fig. 1. Supplemental figure that outlines the strategy and logical process behind these analyses....4

Supplementary Fig. 2. Supplemental LDSC figures for leukocyte telomere length and six cardiovascular diseases....6

Supplementary Fig. 3. Supplemental MiXeR figures for leukocyte telomere length and six cardiovascular diseases 7

Supplementary Fig. 4. The Causal inference between leukocyte telomere length and six cardiovascular diseases....10

Supplementary Fig. 5. Quantile-quantile (Q-Q) plots for PLACO results of leukocyte telomere length and six cardiovascular diseases....12

Supplementary Fig. 6. Locus comparing plots for the shared causal variant for the associations of leukocyte telomere length and six cardiovascular diseases....14

Supplementary Fig. 7. Supplemental plots for functional enrichment analysis targeting tissue-specific genes using ToppGene Functional Annotation tool....21

Supplementary Note 1. Shared genetic architecture contributes to risk of leukocyte telomere length and five major cardiovascular diseases of East Asian....22

Supplementary References. 30

(Source data are provided with this paper)

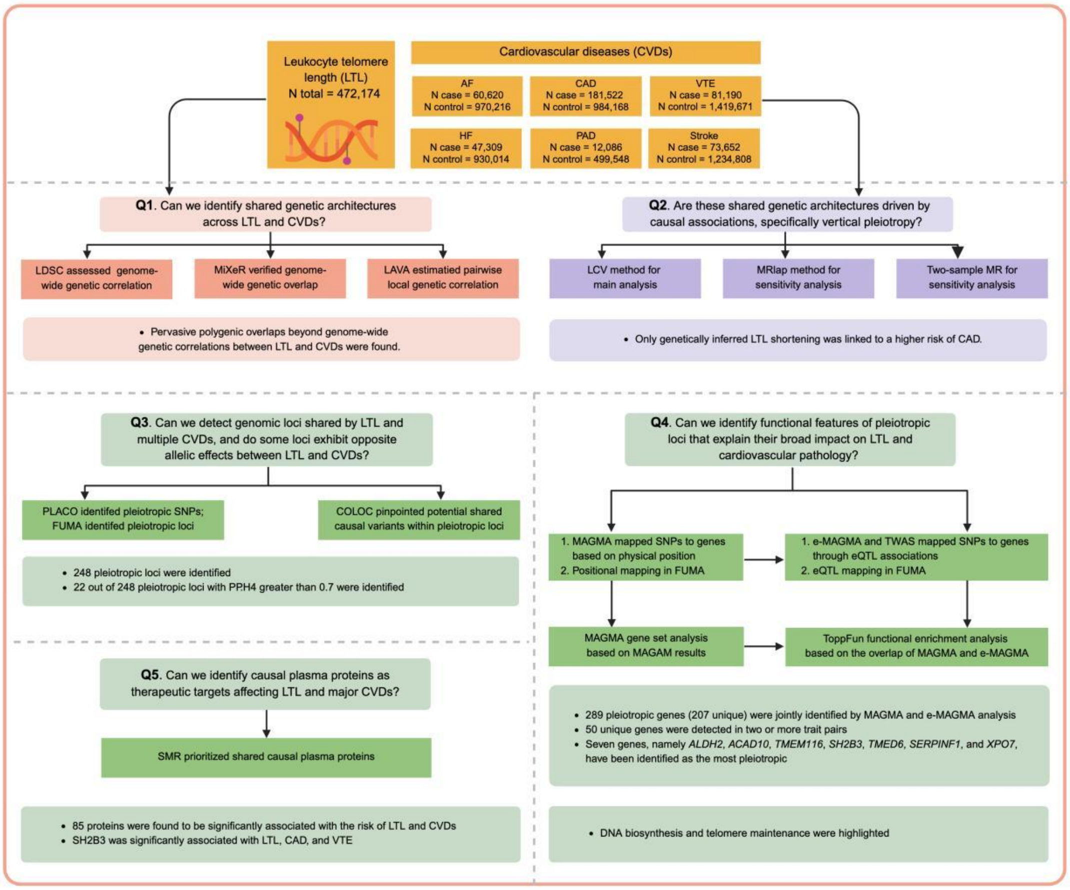

Supplementary Fig. 1. Supplemental figure that outlines the logical strategy, process, and results behind these analyses.

We addressed five central questions regarding the shared genetic basis of LTL and six major CVDs: (i) Can we identify shared genetic architectures across LTL and CVDs? (ii) Are these shared genetic architectures driven by causal associations, specifically vertical pleiotropy? (iii) Can we detect genomic loci shared by LTL and multiple CVDs, and do some loci exhibit opposite allelic effects between LTL and CVDs? (iv) Can we identify functional features of pleiotropic loci that explain their broad impact on LTL and cardiovascular pathology? (v) Can we identify causal plasma proteins as therapeutic targets affecting LTL and major CVDs? The diagram was created using BioRender and included with permission for publication (Created in BioRender. Feng, Y. (2025) https://BioRender.com/4f4qdvh). LTL, leukocyte telomere length; AF, Atrial fibrillation; CAD, Coronary artery disease; VTE, Venous thromboembolism; HF, Heart failure; PAD, Peripheral artery disease. LD, Linkage disequilibrium; LDSC, Linkage disequilibrium score regression; MiXeR, Causal mixture model; LAVA, Local Analysis of

[co]Variant Annotation; LCV, Latent causal variable; MR, Mendelian randomization; PLACO, Pleiotropic analysis under composite null hypothesis; FUMA, Functional Mapping and Annotation; PP.H4, Posterior Probability for Hypothesis 4; MAGMA, Multi-marker Analysis of GenoMic Annotation; e-MAGMA, eQTL-informed MAGMA; TWAS, Transcriptome-wide association study; ToppFun, ToppGene Functional Annotation tool.

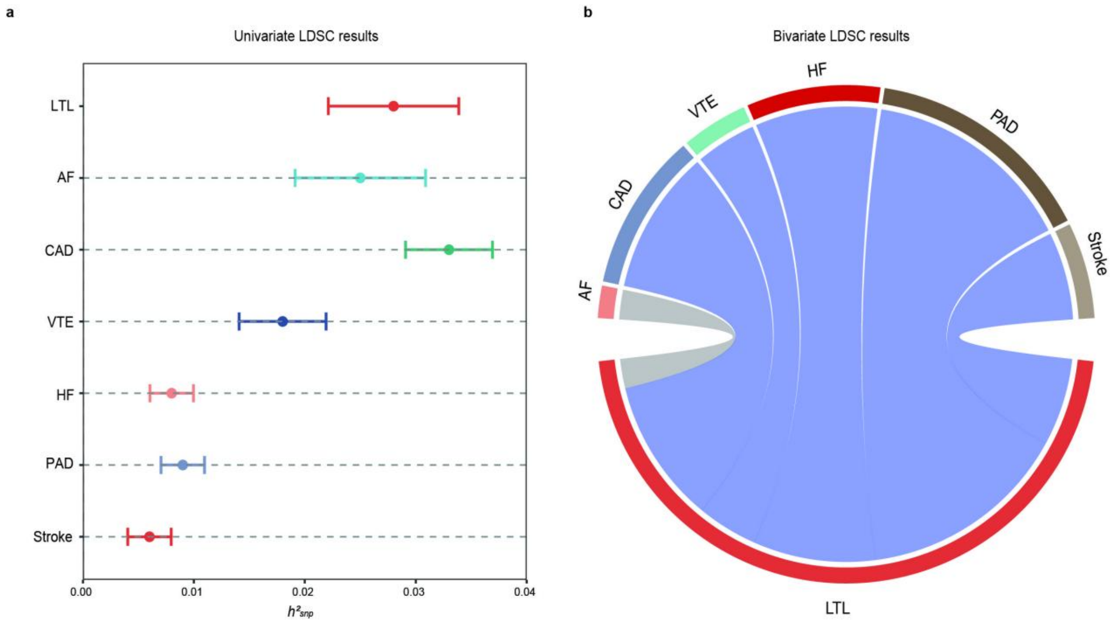

Supplementary Fig. 2. Supplemental LDSC figures for leukocyte telomere length and six cardiovascular diseases.

(a) Error-bar plot of the SNP-based heritability (h^2_{SNP}) point estimates for leukocyte telomere length and six major cardiovascular diseases, computed by univariate LDSC. **(b)** Network visualization of the Bonferroni-corrected significant global r_g between leukocyte telomere length and six major cardiovascular diseases, computed by bivariate LDSC. Connections represent significant r_g, with correlation value along connections, thicker lines denoting stronger correlations. Blue indicates negative correlations and dark gray indicates insignificant correlations. The size of the nodes is weighed by the sample size and h^2_{SNP} of the given phenotype (size = h^2_{SNP} × sqrt(N)). All statistical tests were two-sided. LTL, leukocyte telomere length; AF, Atrial fibrillation; CAD, Coronary artery disease; VTE, Venous thromboembolism; HF, Heart failure; PAD, Peripheral artery disease.

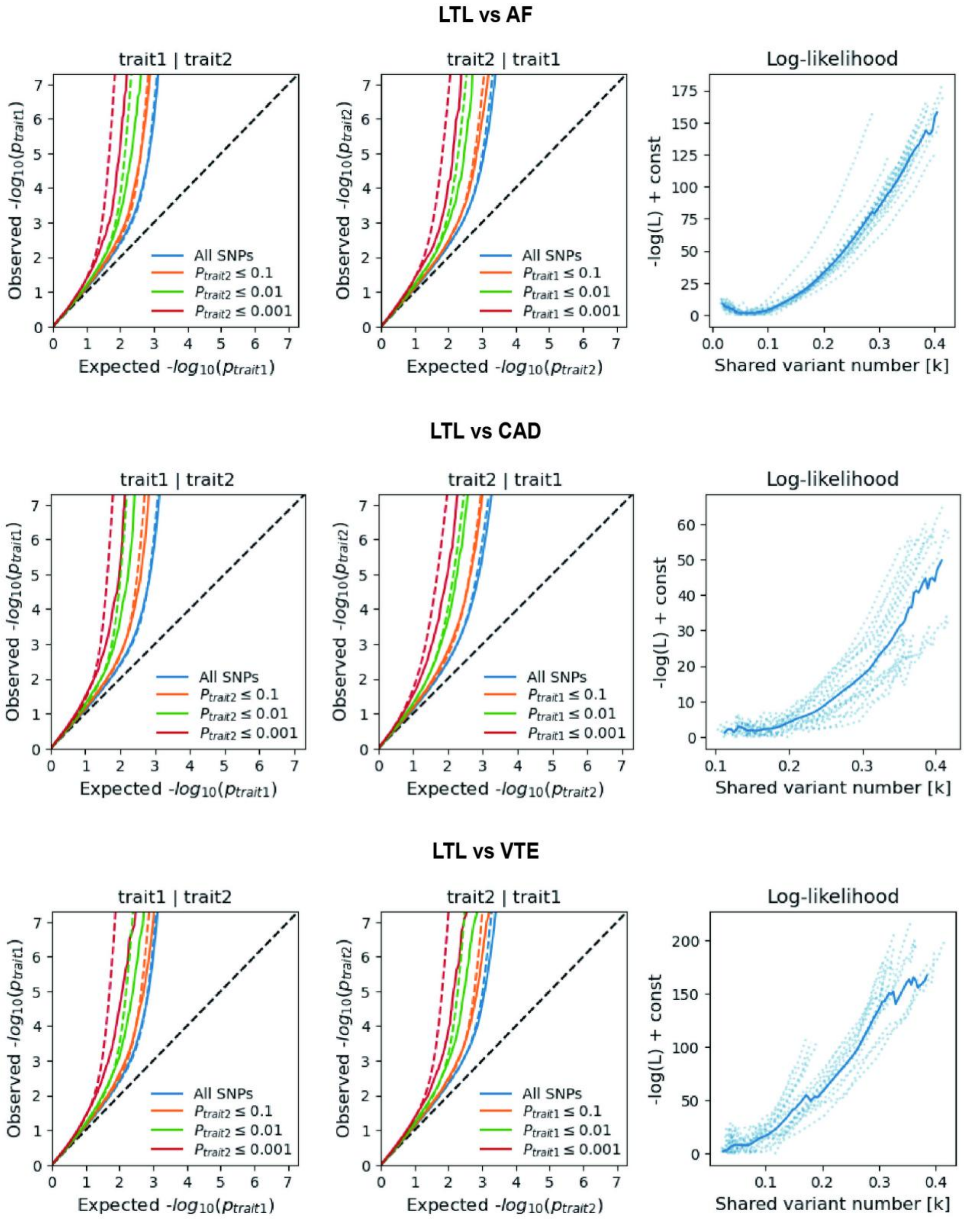
LTL vs AF
trait1 | trait2
trait2 | trait1
Log-likelihood
All SNPs
Observed -log10(ptrait1)
Expected -log10(ptrait1)
Observed -log10(ptrait2)
Expected -log10(ptrait2)
-log(L) + const
Shared variant number [k]
LTL vs CAD
LTL vs VTE

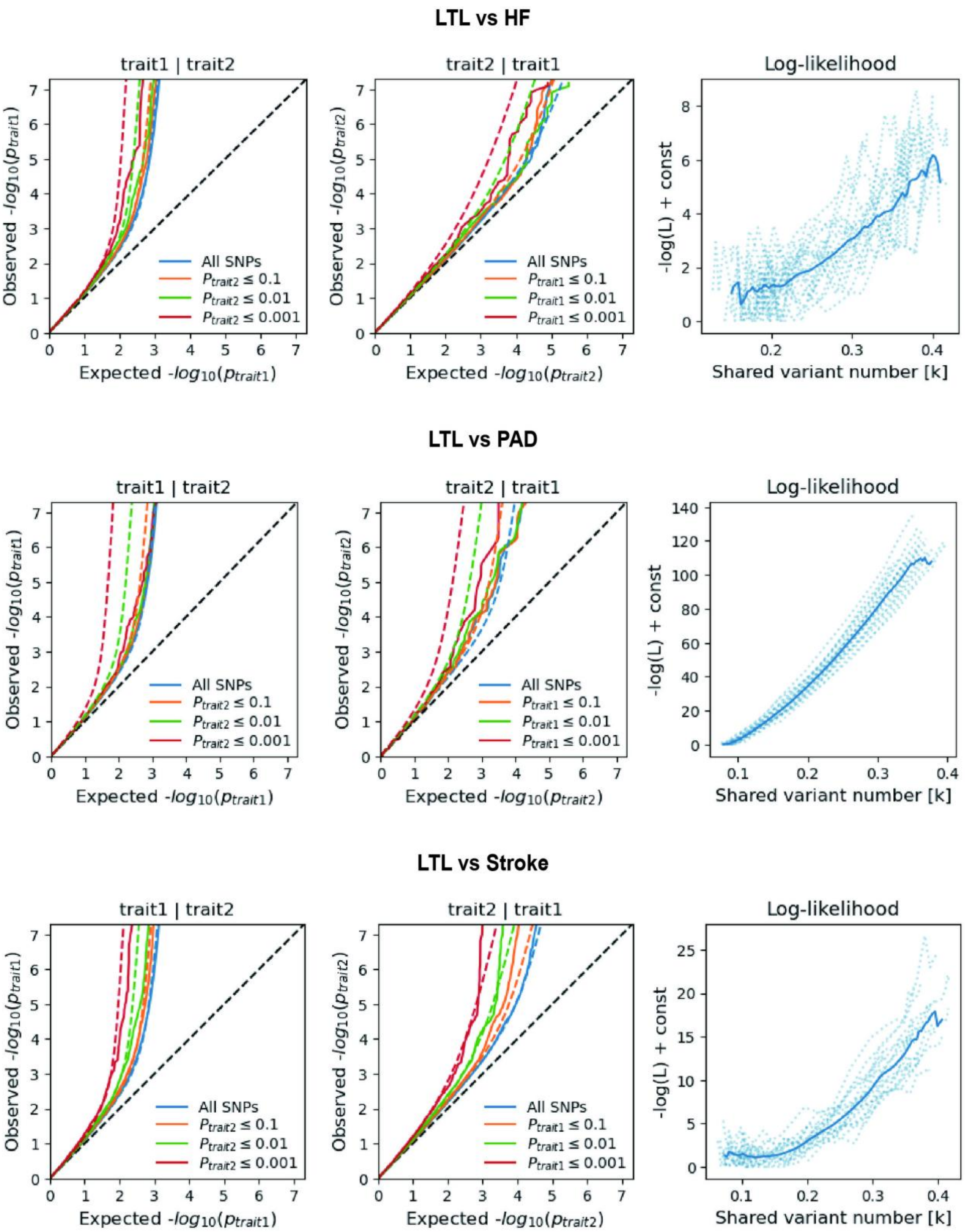
LTL vs HF
trait1 | trait2
trait2 | trait1
Log-likelihood
Observed -$log_{10}(p_{trait1})$
Expected -$log_{10}(p_{trait1})$
Observed -$log_{10}(p_{trait2})$
Expected -$log_{10}(p_{trait2})$
All SNPs
$P_{trait2} \leq 0.1$
$P_{trait2} \leq 0.01$
$P_{trait2} \leq 0.001$
$P_{trait1} \leq 0.1$
$P_{trait1} \leq 0.01$
$P_{trait1} \leq 0.001$
-log(L) + const
Shared variant number [k]
LTL vs PAD
LTL vs Stroke

Supplementary Fig. 3. Supplemental MiXeR figures for each of leukocyte telomere length and six cardiovascular diseases.

On the left, conditional QQ plots show for the observed versus expected -log10 *P*-values of the primary trait, based on the significance of its association with the secondary trait across all SNPs (blue lines)， $P \leq 0.1$ (orange lines), $P \leq 0.01$ (green lines), and $P \leq 0.001$ (red lines). Dotted lines represent model predictions for each stratum. The black dotted line represents the expected Q-Q plot under the null hypothesis (no SNPs are associated with the phenotype). The points on the Q-Q plot were weighted according to LD structure, using 64 iterations of random pruning at an LD threshold $r^2 = 0.1$. **On the right**, log-likelihood curves illustrate the goodness of model fit by plotting the negative log-likelihood function (lower values correspond to better model fit) against the π_{12} parameter (the number of influencing variants shared between two traits). The remaining parameters of the model were fixed at their estimated values. The π_{12} range on the log-likelihood plots spans from the smallest possible value $\pi_{12} = r_g$*sqrt(π_{1u}, π_{2u}) that was still compatible with the estimated genetic correlation, up to the largest possible value $\pi_{12} = \min(\pi_{1u}, \pi_{2u})$ that corresponded to the minimum total polygenicity among the two traits. The minimum point represents the best-fitting model estimate of the number of influencing variants shared between the two traits. All statistical tests were two-sided. LTL, leukocyte telomere length; AF, Atrial fibrillation; CAD, Coronary artery disease; VTE, Venous thromboembolism; HF, Heart failure; PAD, Peripheral artery disease.

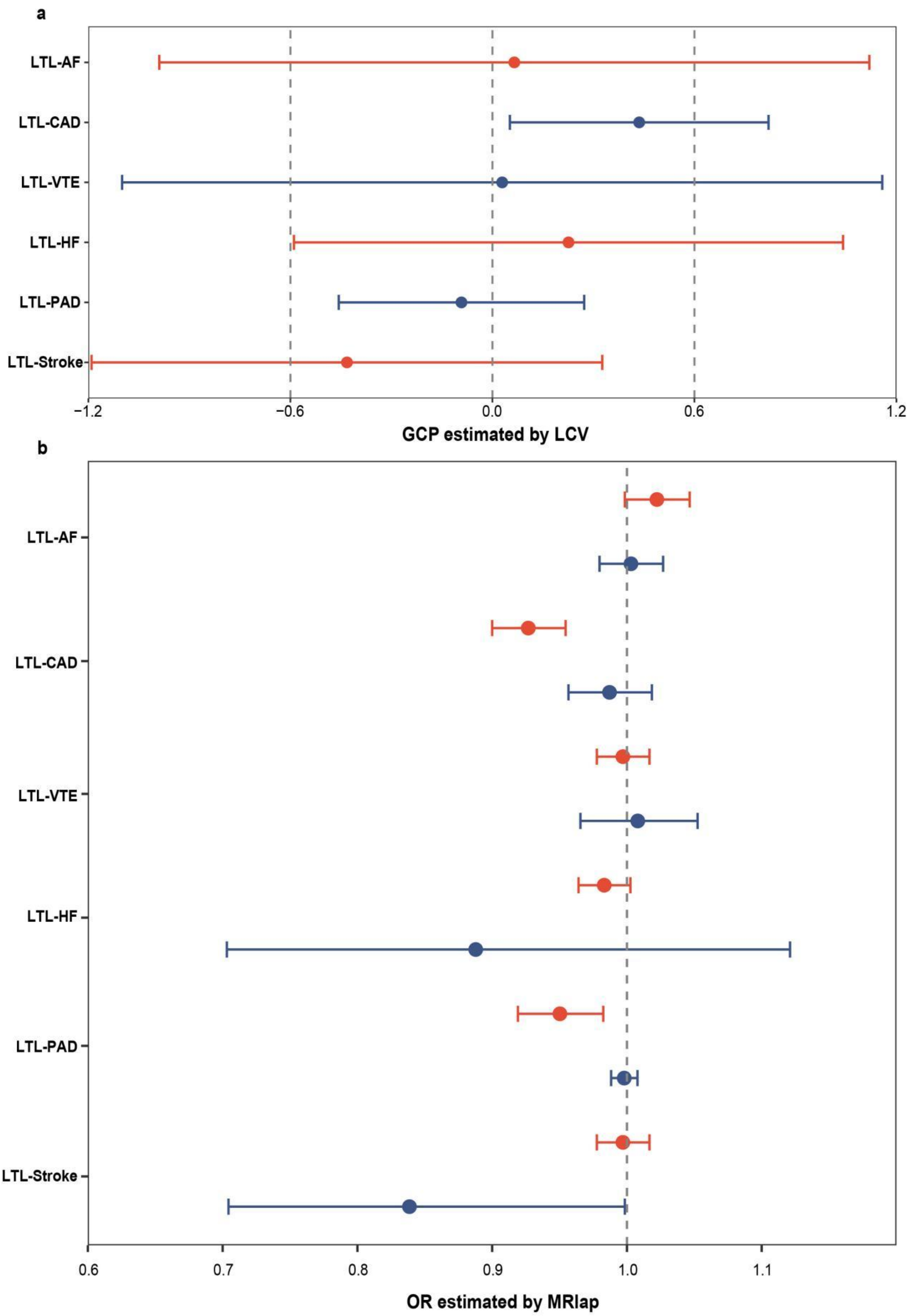

Supplementary Fig. 4. The causal inference between leukocyte telomere length and six cardiovascular diseases.

Summary of putative causal relationships between LTL and six CVDs identified by LCV (a) and MRlap (b). **(a)** Forest plot of the LCV analysis on the associations between LTL and six CVDs.

Circles represent the genetic causality proportion (GCP) estimate, and the error bars indicate standard errors (SE). GCP and SE_{GCP} are posterior mean genetic causality proportions and posterior standard errors estimated by the LCV model. **(b)** Forest plot of the MRlap analysis on the association between LTL and six CVDs. Circles represent the odd ratio (OR) estimate, and the error bars indicate the 95% confidence interval. Results colored in red represent the estimated causal effect of LTL on CVDs, while results colored in blue represent the estimated causal effect of CVDs on LTL. A positive association is indicated by OR > 1, while a negative association is indicated by OR < 1. All statistical tests were two-sided. LTL, leukocyte telomere length; AF, Atrial fibrillation; CAD, Coronary artery disease; VTE, Venous thromboembolism; HF, Heart failure; PAD, Peripheral artery disease.

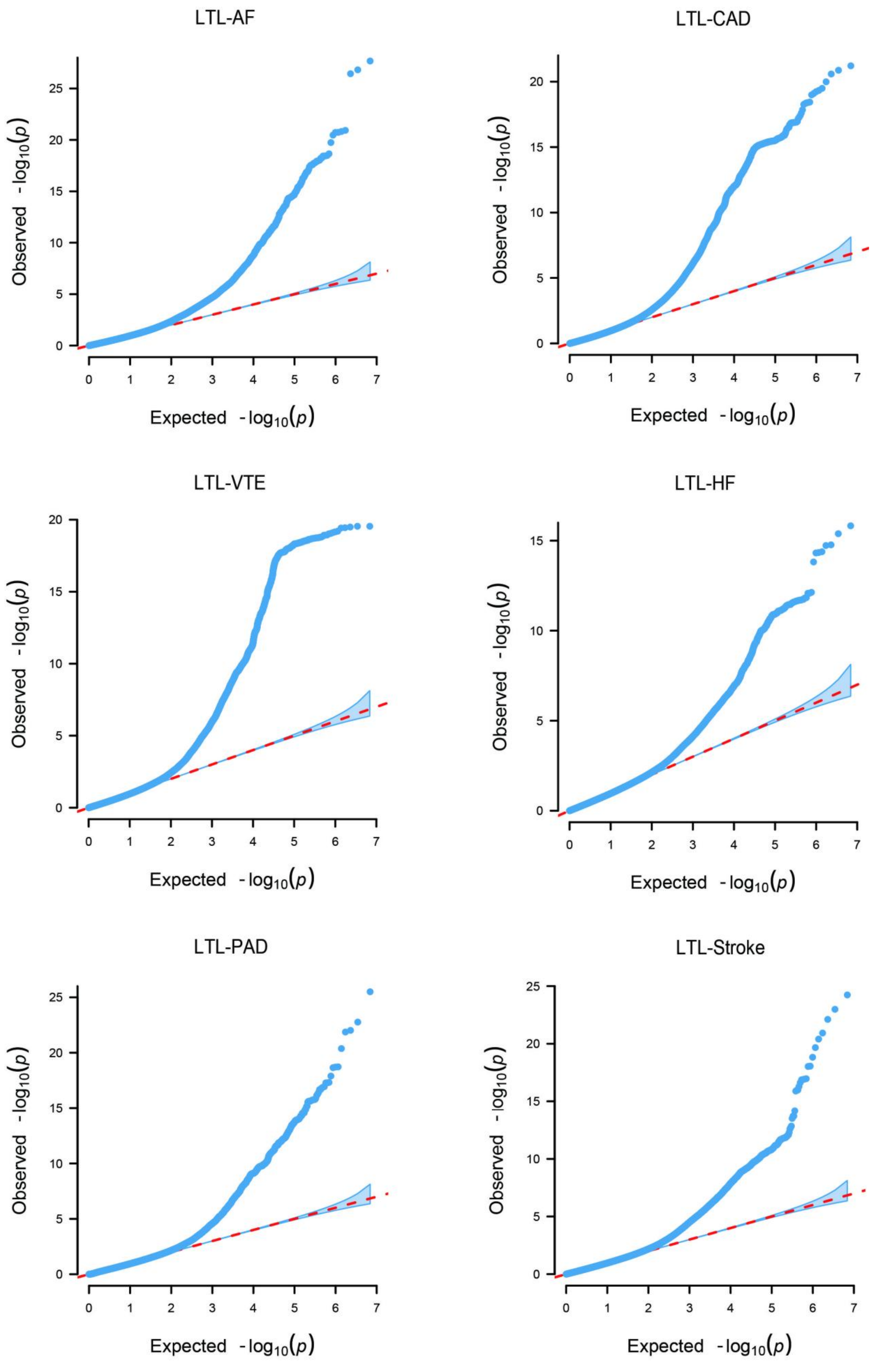
LTL-AF
LTL-CAD
LTL-VTE
LTL-HF
LTL-PAD
LTL-Stroke
Observed -log$_{10}$(p)
Expected -log$_{10}$(p)

Supplementary Fig. 5. Quantile-quantile (Q-Q) plots for PLACO results of leukocyte telomere length and six cardiovascular diseases.

Q-Q plots depicts expected -log10 *P*-values (*x*-axis) against observed -log10 P_{PLACO}-values (*y*-axis). Red dots indicate significant pleiotropic variants ($P_{PLACO} < 5\times10^{-8}$). All statistical tests were two-sided. LTL, leukocyte telomere length; AF, Atrial fibrillation; CAD, Coronary artery disease; VTE, Venous thromboembolism; HF, Heart failure; PAD, Peripheral artery disease.

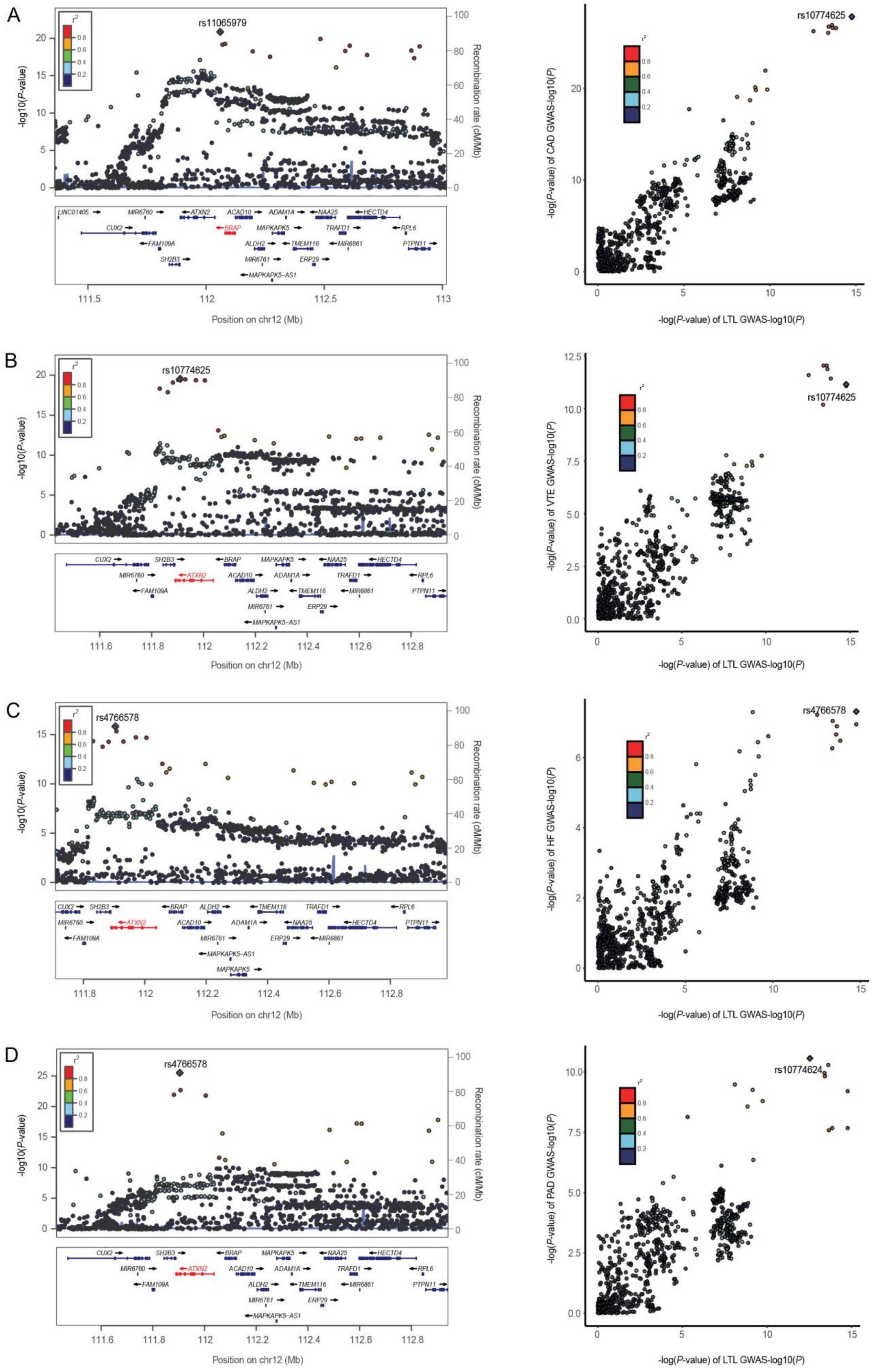
A
rs11065979
-log10(P-value)
Recombination rate (cM/Mb)
Position on chr12 (Mb)
LINC01405
CUX2
FAM109A
SH2B3
MIR6760
ATXN2
BRAP
ACAD10
ALDH2
MIR6761
MAPKAPK5-AS1
ADAM1A
MAPKAPK5
TMEM116
ERP29
NAA25
TRAFD1
MIR6861
HECTD4
RPL6
PTPN11
rs10774625
-log(P-value) of CAD GWAS-log10(P)
-log(P-value) of LTL GWAS-log10(P)
B
rs10774625
-log(P-value) of VTE GWAS-log10(P)
C
rs4766578
-log(P-value) of HF GWAS-log10(P)
D
rs4766578
rs10774624
-log(P-value) of PAD GWAS-log10(P)

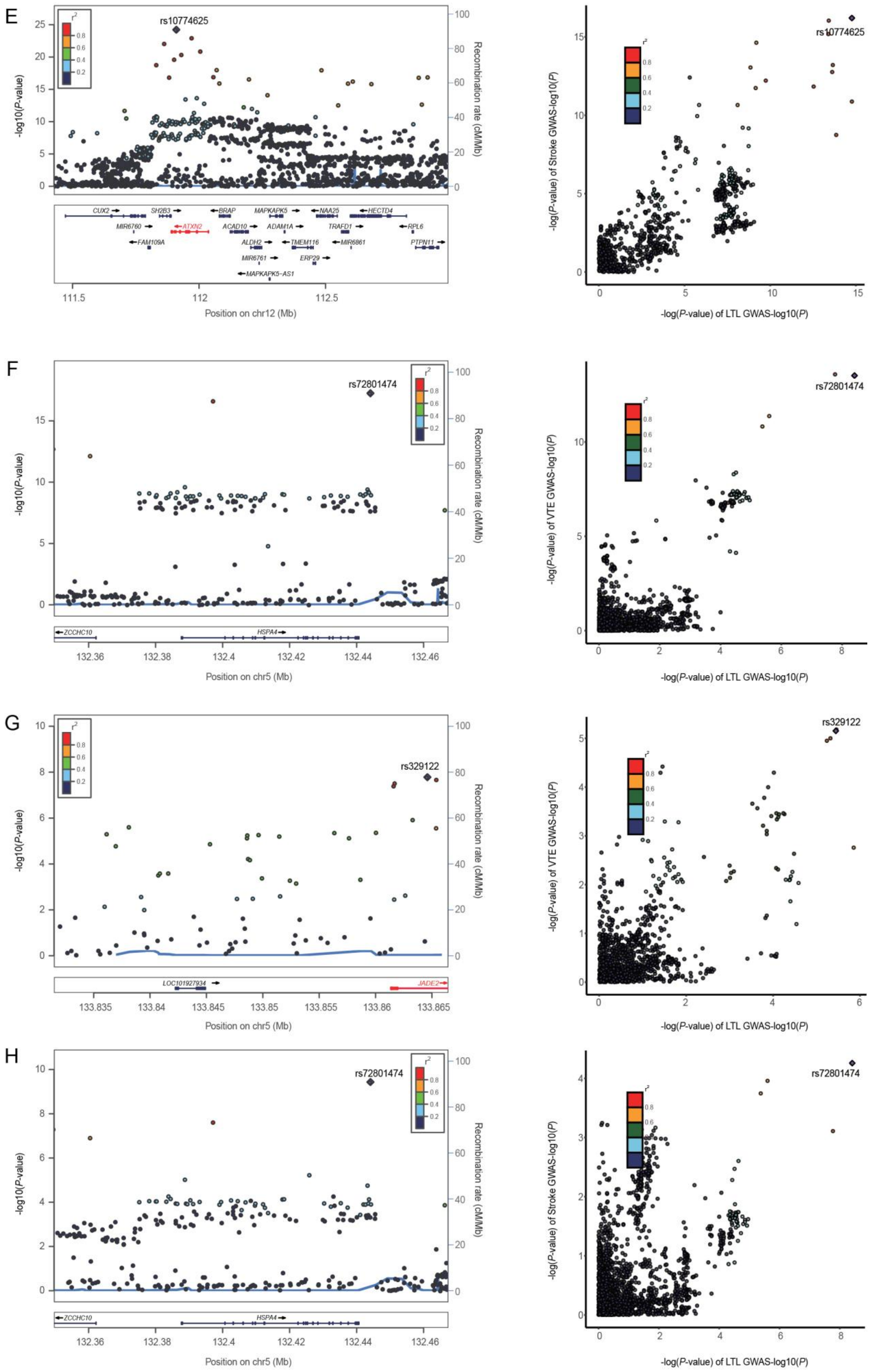
E
rs10774625
-log10(P-value)
Recombination rate (cM/Mb)
CUX2
SH2B3
BRAP
MAPKAPK5
NAA25
HECTD4
MIR6760
ATXN2
ACAD10
ADAM1A
TRAFD1
RPL6
FAM109A
ALDH2
TMEM116
MIR6861
PTPN11
MIR6761
ERP29
MAPKAPK5-AS1
Position on chr12 (Mb)
-log(P-value) of Stroke GWAS-log10(P)
-log(P-value) of LTL GWAS-log10(P)
F
rs72801474
ZCCHC10
HSPA4
Position on chr5 (Mb)
-log(P-value) of VTE GWAS-log10(P)
G
rs329122
LOC101927934
JADE2
H
rs72801474
ZCCHC10
HSPA4
Position on chr5 (Mb)
-log(P-value) of Stroke GWAS-log10(P)
-log(P-value) of LTL GWAS-log10(P)

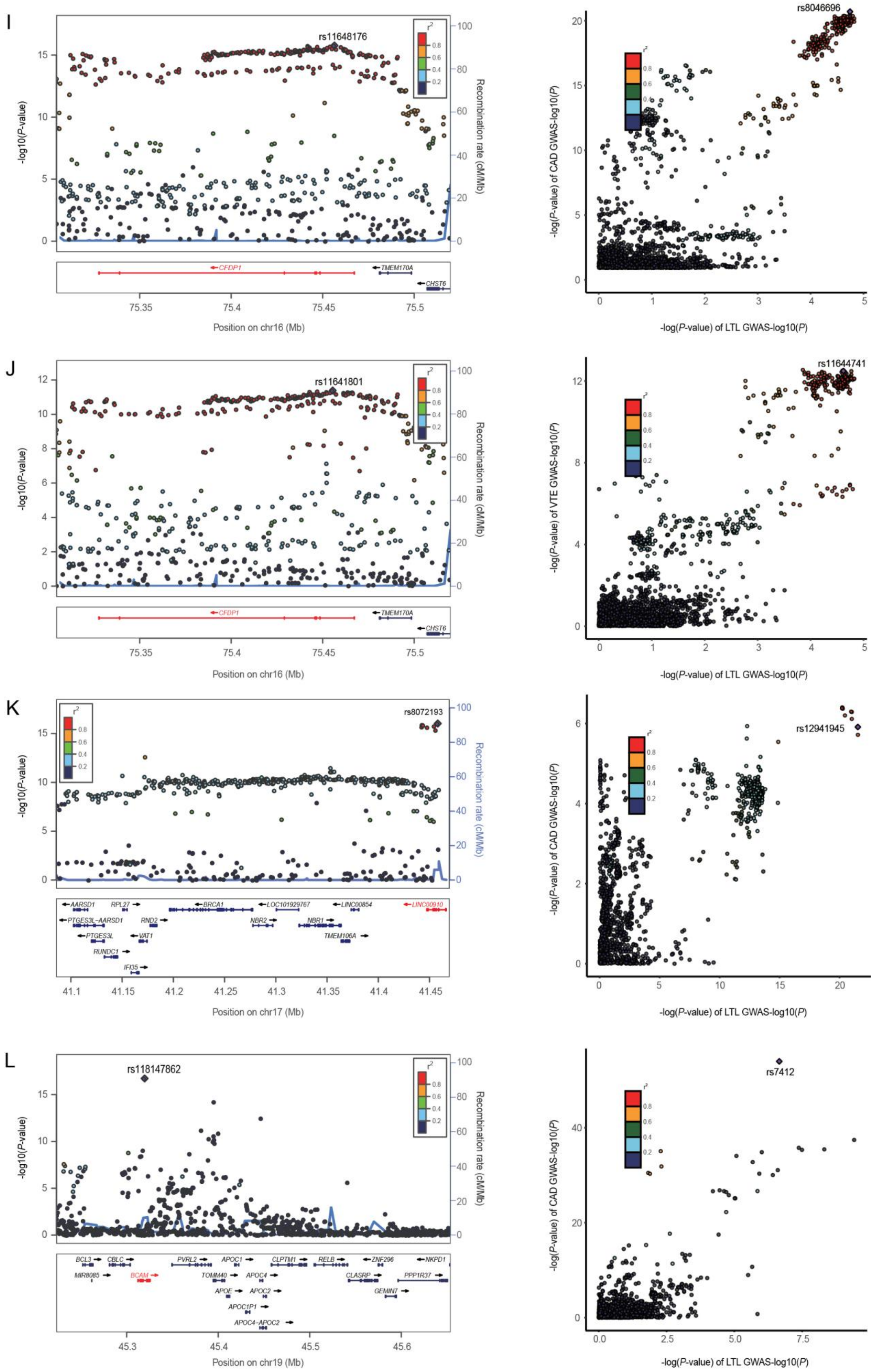

I
rs11648176
-log10(P-value)
Recombination rate (cM/Mb)
CFDP1
TMEM170A
CHST6
Position on chr16 (Mb)
rs8046696
-log(P-value) of CAD GWAS-log10(P)
-log(P-value) of LTL GWAS-log10(P)
J
rs11641801
-log10(P-value)
Recombination rate (cM/Mb)
CFDP1
TMEM170A
CHST6
Position on chr16 (Mb)
rs11644741
-log(P-value) of VTE GWAS-log10(P)
-log(P-value) of LTL GWAS-log10(P)
K
rs8072193
-log10(P-value)
Recombination rate (cM/Mb)
AARSD1
RPL27
BRCA1
LOC101929767
LINC00854
LINC00910
PTGES3L-AARSD1
RND2
NBR2
NBR1
PTGES3L
VAT1
TMEM106A
RUNDC1
IFI35
Position on chr17 (Mb)
rs12941945
-log(P-value) of CAD GWAS-log10(P)
-log(P-value) of LTL GWAS-log10(P)
L
rs118147862
-log10(P-value)
Recombination rate (cM/Mb)
BCL3
CBLC
PVRL2
APOC1
CLPTM1
RELB
ZNF296
NKPD1
MIR8085
BCAM
TOMM40
APOC4
CLASRP
PPP1R37
APOE
APOC2
GEMIN7
APOC1P1
APOC4-APOC2
Position on chr19 (Mb)
rs7412
-log(P-value) of CAD GWAS-log10(P)
-log(P-value) of LTL GWAS-log10(P)

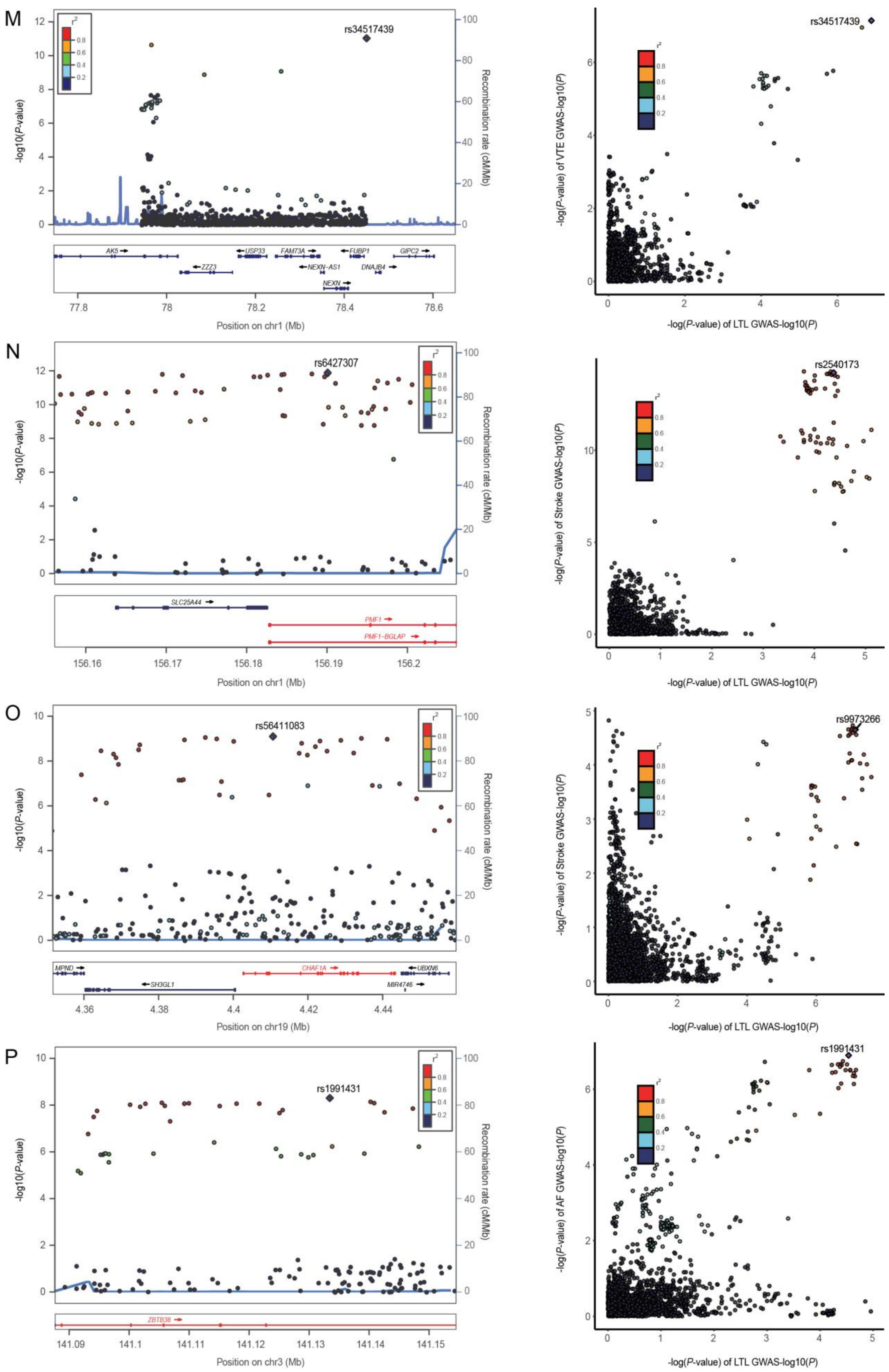
M
rs34517439
-log10(P-value)
Recombination rate (cM/Mb)
AK5
ZZZ3
USP33
FAM73A
NEXN-AS1
NEXN
FUBP1
DNAJB4
GIPC2
Position on chr1 (Mb)
-log(P-value) of VTE GWAS-log10(P)
-log(P-value) of LTL GWAS-log10(P)
N
rs6427307
SLC25A44
PMF1
PMF1-BGLAP
Position on chr1 (Mb)
rs2540173
-log(P-value) of Stroke GWAS-log10(P)
O
rs56411083
MPND
SH3GL1
CHAF1A
UBXN6
MIR4746
Position on chr19 (Mb)
rs9973266
P
rs1991431
ZBTB38
Position on chr3 (Mb)
-log(P-value) of AF GWAS-log10(P)

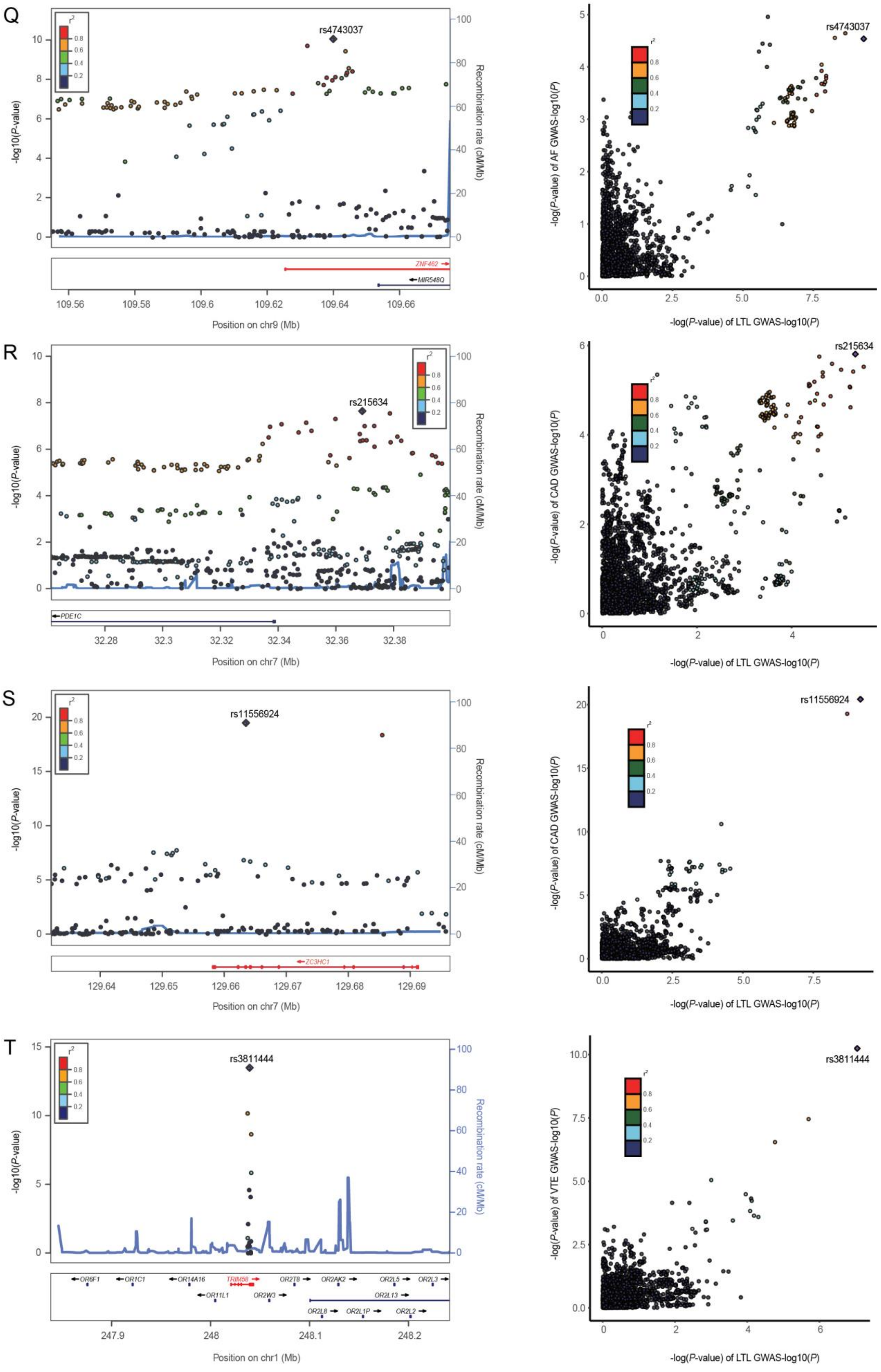
Q
rs4743037
-log10(P-value)
Recombination rate (cM/Mb)
ZNF462
MIR548Q
Position on chr9 (Mb)
-log(P-value) of AF GWAS-log10(P)
-log(P-value) of LTL GWAS-log10(P)
R
rs215634
PDE1C
Position on chr7 (Mb)
-log(P-value) of CAD GWAS-log10(P)
S
rs11556924
ZC3HC1
Position on chr7 (Mb)
T
rs3811444
OR6F1
OR1C1
OR14A16
TRIM58
OR2T8
OR2AK2
OR2L5
OR2L3
OR11L1
OR2W3
OR2L13
OR2L8
OR2L1P
OR2L2
Position on chr1 (Mb)
-log(P-value) of VTE GWAS-log10(P)

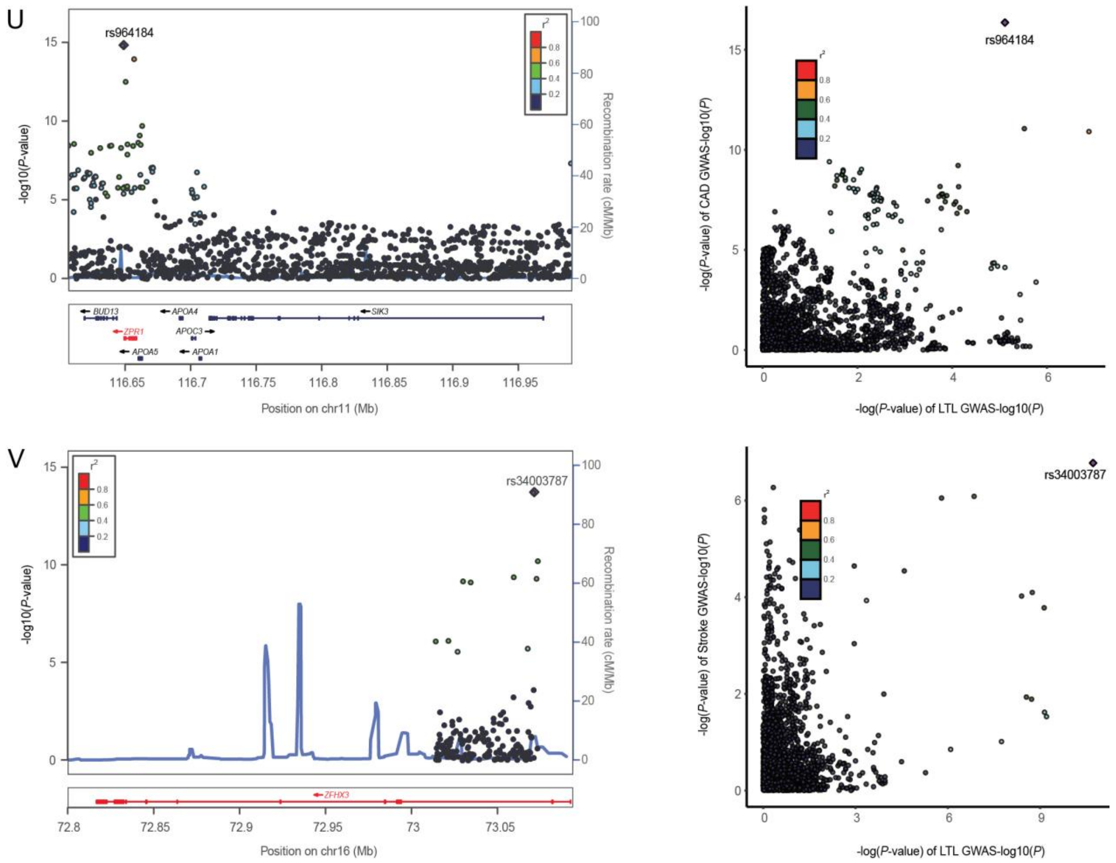

Supplementary Fig. 6. Locus comparing plots for the shared causal variant for the associations of leukocyte telomere length and six cardiovascular diseases.

The top 22 genomic loci are influencing LTL and CVDs via shared SNPs. For each colocalized locus (PP.H4 > 0.7) identified for the corresponding trait pair, the left panel depicts the PLACO results using a LocusZoom plot, and the right panel compares two single-trait GWAS statistics of the corresponding trait pair for each variant using LocusCompare plot. For the LocusZoom plot, the *x*-axis shows the genomic position for each variant, and the *y*-axis shows -log10 *P* values from PLCAO results. The top variant with the smallest P_{PLACO} in each locus is indicated in purple diamond. The color of each variant represents its LD relationship with the top variant. For the LocusCompare plot, each dot represents a variant; the *x*-axis shows the -log10 P_{GWAS} from the corresponding GWAS of LTL, and the *y*-axis shows -log10 P_{GWAS} from the corresponding GWAS trait. A purple diamond also indicates the candidate-shared causal variant identified by pairwise colocalization analysis. The color of each variant represents its LD relationship with the candidate-shared causal variant. All genomic locations are based on reference genome hg19, and the LD calculation is based on the 1000 Genomes Project of the European population. (A)

12q24.12 for LTL-CAD; (B) 12q24.12 for LTL-VTE; (C) 12q24.12 for LTL-HF; (D) 12q24.12 for LTL-PAD; (E) 12q24.12 for LTL-stroke; (F) 5q31.1 for LTL-VTE (rs72801474); (G) 5q31.1 for LTL-VTE (rs329122); (H) 5q31.1 for LTL-stroke; (I) 16q23.1 for LTL-CAD; (J) 16q23.1 for LTL-VTE; (K) 17q21.31 for LTL-CAD; (L) 19q13.32 for LTL-CAD; (M) 1p31.1 for LTL-VTE; (N) 1q22 for LTL-stroke; (O) 19p13.3 for LTL-stroke; (P) 3q23 for LTL-AF; (Q) 9q31.2 for LTL-AF; (R) 7p14.3 for LTL-CAD; (S) 7q32.2 for LTL-CAD; (T) 1q44 for LTL-VTE; (U) 11q23.3 for LTL-CAD; (V) 16q22.3 for LTL-stroke. Detailed descriptions were provided in Supplementary Data 7. All statistical tests were two-sided. LTL, leukocyte telomere length; AF, Atrial fibrillation; CAD, Coronary artery disease; VTE, Venous thromboembolism; HF, Heart failure; PAD, Peripheral artery disease.

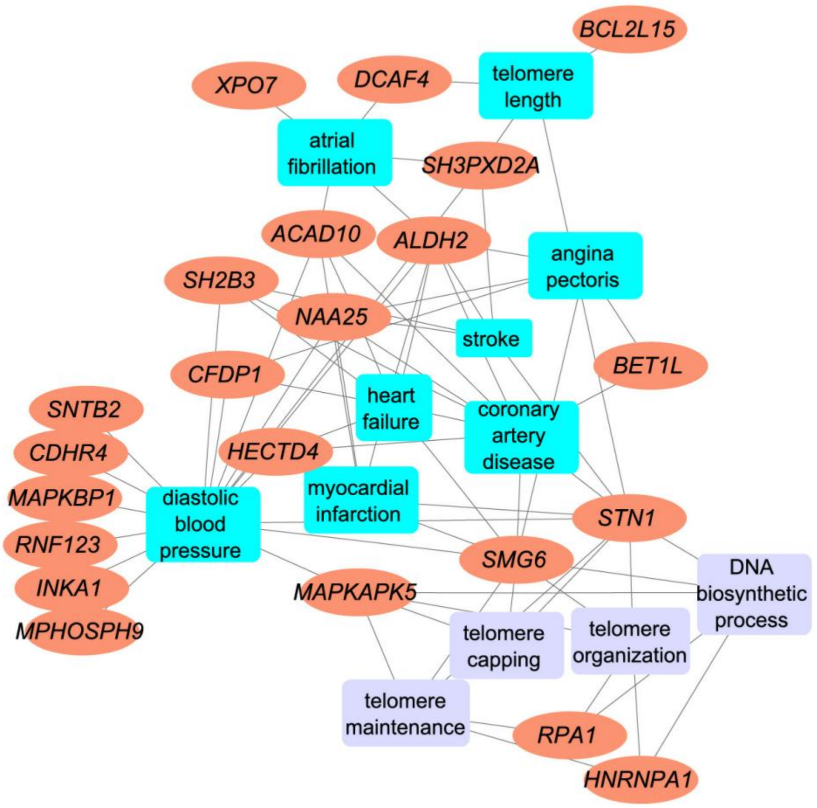

Supplementary Fig. 7. Supplemental plots for functional enrichment analysis targeting tissue-specific genes using ToppGene Functional Annotation tool.

A connectivity network that reveals functional features shared to multiple gene lists. The ToppFun functional enrichment analyses (FEA) concerning diseases and pathways validated that 22 genes are associated with LTL and CVD-related traits. The four significantly enriched annotations were highlighted, namely ‘telomere maintenance,’ ‘telomere organization,’ ‘telomere capping,’ and ‘DNA biosynthesis process.’ All statistical tests were two-sided. LTL, leukocyte telomere length; AF, Atrial fibrillation; CAD, Coronary artery disease; VTE, Venous thromboembolism; HF, Heart failure; PAD, Peripheral artery disease.

Supplementary Note 1. Shared genetic architecture contributes to risk of leukocyte telomere length and five major cardiovascular diseases of East Asian.

Section 1. Data Sources and Quality Control of East Asian Ancestry

Analyzing GWAS summary statistics from multiple ancestries reveals a more comprehensive genetic landscape, capturing global diversity and offering an advantage for discovering genetic signals that failed to be identified in European populations. Despite numerous genetic studies on LTL or CVDs in non-European populations, these typically feature smaller sample sizes than those focusing on European populations. However, Dorajoo et al. reported a GWAS of LTL in a relatively large Singaporean East-Asian (Southern Han Chinese) ethnic population (n = 23,096)[1]. Both LTL GWAS from UKB and Singapore Chinese Health Study (SCHS) were derived from blood samples collected at baseline and telomere length values were determined using a relative ratio of telomere repeat copy number to a single copy gene (T/S) based on single-color quantitative polymerase chain reaction (qPCR) after adjusting for the technical parameters (i.e., enzyme batch, temperature, operator, primer batch, and humidity). Ishigaki et al. reported a comprehensive GWAS on 42 common diseases conducted through the BioBank Japan Project (BBJ), one of the largest non-European biobanks with approximately 200,000 Japanese individuals. This study included analyses of four major CVDs (CAD, HF, PAD, and stroke)[2]. Concurrently, Low et al. conducted the largest GWAS on AF in the Japanese population, involving 8,180 cases and 28,612 controls[3]. Zhang et al. conducted the first GWAS on VTE in a Han Chinese cohort comprising 1,268 cases and 17,663 controls[4]. As of the latest analysis, GWAS summary statistics for VTE remain unpublished. Finally, we assembled the most extensive available GWAS datasets for five major CVDs in East Asian individuals: AF, CAD, HF, PAD, and stroke. Notably, the sample size for the AF GWAS did not exceed 50,000. Detailed information on these diseases and their publication sources is provided in Supplementary Data 20. These GWAS summary data were aligned with the hg19 genome build, referencing the 1000 Genomes Project Phase 3 East Asian. Further details regarding our quality control procedures are elaborated in the main text, ensuring rigorous standards in data analysis.

Section 2. Genetic overlap beyond genetic correlation between LTL and five major CVDs of East Asian ancestry

We employed cross-trait linkage disequilibrium (LD) score regression (LDSC) to calculate SNP-based heritability (h^2_{SNP}) and to assess genome-wide genetic correlation (r_g) between LTL and five major CVDs. This analysis utilized pre-computed LD scores from the East Asian-based reference panel in the 1000 Genomes Project Phase 3, and SNPs that did not intersect the reference panel were removed from the LDSC analysis. Genetic correlations with P-values below the Bonferroni-adjusted threshold (P = 0.05 / number of trait pairs = 0.05 / 5 = 0.01) were deemed statistically significant. Univariate LDSC revealed that the estimated h^2_{SNP} for LTL was 0.138 (SE = 0.025). The strength of the genetic signals of GWASs varied considerably among the five CVDs, with the h^2_{SNP} ranging from 0.010 to 0.144 (Supplementary Data 21a). The estimated h^2_{SNP} was higher for AF (h^2_{SNP} = 0.144, SE = 0.032) and CAD (h^2_{SNP} = 0.070, SE = 0.007), in comparison to HF (h^2_{SNP} = 0.010, SE = 0.002), PAD (h^2_{SNP} = 0.015, SE = 0.003), and stroke (h^2_{SNP} = 0.015, SE = 0.003). The results exhibited a notable level of congruity with the findings of the European population. Consistent with findings in European populations, bivariate LDSC analysis detected the most pronounced negative genetic correlation between LTL and PAD (r_g = -0.245, P = 0.047; Supplementary Data 21b) but fell short of the stringent Bonferroni threshold. No significant genome-wide genetic correlations between LTL and other CVDs were observed, potentially due to the limited sample size and the exclusion of Major Histocompatibility Complex (MHC) regions in the LDSC framework, which may have affected the outcomes.

Although limited genome-wide genetic correlations were observed between LTL and CVDs, this may be attributed to local genetic correlations of opposing directions, which attenuate correlations at the genome-wide level and potentially underestimate the genetic overlap. Additionally, the polygenic nature of LTL could obscure stronger genetic associations with CVD.

MiXeR quantifies polygenic overlap beyond genetic correlation by estimating the total number of both shared and trait-specific causal variants (i.e., variants with non-zero additive genetic effects on a given trait), irrespective of genetic correlation using GWAS summary statistics, and is agnostic to effect directions. Univariate MiXeR revealed that LTL was less polygenic (N = 31, SD = 19). Among the five major CVDs, HF (N = 1,516 ‘causal’ variants explaining 90% of HF’s h^2_{SNP}, SD = 562; Supplementary Data 22a) demonstrated the highest polygenicity, followed by PAD (N

= 954, SD = 211), and stroke (N = 871, SD = 124). AF (N = 29, SD = 7) and CAD (N = 630, SD = 42) showed lower polygenicity. The large standard deviations for the polygenicity estimates for HF and PAD indicate that these estimates should be interpreted cautiously, likely reflecting a combination of low SNP-heritability and insufficient GWAS power for these disorders.

Bivariate MiXeR results showed that a substantial proportion of the genetic variants associated with LTL also affect CVDs, but the number of shared and trait-specific variants and the balance of protective and risk-enhancing variants differ across trait pairs (Supplementary Data 22b). Causal variants exhibited distinct patterns of polygenic overlap between LTL and CVDs. LTL had similar levels of polygenicity and a more significant degree of polygenic overlap with AF, CAD, and stroke. For example, we discovered LTL and AF shared 5 (SD = 2) out of 55 variants with particularly striking polygenic overlap (Dice coefficient = 0.175, SD = 0.096). The shared variants represent 15.63% of the genetic variants influencing LTL and 16.84% of the variants underlying AF. MiXeR estimated that of the 31 LTL-influencing variants, 57.50% and 66.59% also influence CAD and stroke, respectively. The observed extensive genetic overlap in the Venn diagrams with almost nonexistent genetic correlations suggests a balanced mixture of concordant and discordant genetic effects across shared loci between LTL and these CVD phenotypes (including AF, CAD, and stroke), with risk variants that did overlap showing low degree of effect direction concordance (34.48%-53.86% of shared variants in the same direction). Given the low polygenicity of LTL compared to highly polygenic diseases such as PAD and HF, substantial differences were observed in the number of shared and unique 'causal' variants. For example, LTL and PAD shared the most significant numbers of ‘causal’ variants (N = 31, SD = 19), with few unique LTL variants (N = 3.22×10^{-3}K, SD = 4.29×10^{-3}K) and many more unique variants of PAD (N = 923, SD = 202), representing 99.99% of the genetic variants influencing LTL and 3.27% of the variants underlying PAD. While they were moderately correlated at the genome-wide level (r_g = -0.177, SE = 0.036), shared variants were strongly correlated ($r_g s$ = -1.000, SE = 1.16×10^{-5}). A similar relationship was evident between LTL and HF. There was complete genetic overlap between LTL and PAD (Dice coefficient = 0.063, SD = 0.026) or HF (Dice coefficient = 0.042, SD = 0.024), potentially attributable to the fact that the GWAS of PAD and HF have a substantially more significant effect on sample size than the GWASs of the other CVDs. However, the AIC values were all negative,

indicating a poor model fit, and thus, this particular analysis is unreliable.

LAVA estimates local SNP heritability and genetic correlations (loc-r_gs) across 1,445 semi-independent genetic regions of approximately 1.8 Mb. It identifies shared genetic regions with their effect directions despite negligible genome-wide r_g. A total of 126 pairwise correlations across regions with adequate univariate signals were performed, which yielded 22 genomic regions with nominally significant correlations in at least one trait pair at $P < 0.05$ (Supplementary Data 23-24). These results provide further evidence of pleiotropy with mixed effect direction among LTL-AF (5 positively correlated and 4 negatively correlated loci), LTL-CAD (2 positively correlated and 2 negatively correlated loci), LTL-HF (1 positively correlated and 1 negatively correlated loci), and LTL-stroke (4 positively correlated and 3 negatively correlated loci). After correcting for multiple testing using FDR (FDR < 0.05), five out of 22 local genomic regions were found to be significant for bivariate analysis, and no unique region is shared between LTL and more than one CVD (Supplementary Data 24). Only two significant local genetic correlations were identified between LTL and AF (LD block 670 on chromosome 7, ranging from 69,083,425 to 71,874,126; LD block 1,060 on chromosome 12, ranging from 114,181,110 to 116,184,485). Three regions (LD block 912 [chr10: 102,471,757-106,139,565], LD block 1,233 [chr16: 25,725,417-27,795,175], and LD block 1,294 [chr17: 69,546,300-72,789,312]) identified between LTL and stroke exhibited a discordant direction of effect. There were few loci with significant local correlations after FDR < 0.05 due to the smaller sample size and lower heritability of LTL, including no significant loci with CAD, HF, and PAD.

While there was no evidence in this study using the LDSC that significant genome-wide genetic correlations exist between LTL and CVDs, the evidence of extensive genetic overlap verified by MiXeR coupled with almost nonexistent genetic correlation reflects shared genetic etiologies with mixed effect directions, a finding further corroborated by the local genetic correlation using LAVA. Significant local estimates of positive and negative genetic correlation between LTL and CVDs observed for several LD blocks suggest a complex interrelationship between these phenotypes that may exist and warrant additional study.

Section 3. The causal inference between LTL and five major CVDs of East Asian ancestry

We evaluated the potential causal relationship between LTL and five major CVDs (i.e., vertical pleiotropy) using LCV analysis. We found strong evidence of a potential causal relationship between LTL and CAD (GCP = 0.676, SE = 0.211, $P = 3.83\times10^{-5}$; Supplementary Data 25a), suggesting that telomere shortening could have a deleterious effect on CAD and implying that their relationship is potentially mediated by vertical pleiotropy. There was insufficient evidence to support a causal relationship between LTL and other CVDs, suggesting the absence of vertical pleiotropy. Furthermore, we observed a high degree of consistency between the MRlap and LCV results. The MRlap analysis reaffirmed the previously identified negative causal association between LTL and CAD (OR = 0.943, 95% CI: 0.913-0.974; Supplementary Data 25b). However, genetically instrumented shorter LTL was not associated with other CVDs, suggesting that the shared genetic basis between LTL and CVDs cannot be fully explained by vertical pleiotropy.

Section 4. Pleiotropic genomic loci identified for LTL and CVDs of East Asian ancestry

In addition to the approaches mentioned above, identifying pleiotropic genetic variants or loci responsible for genome-wide genetic correlations enables further exploration of the genetic overlap and shared etiology between these two types of diseases. We employed PLACO to identify genetic variants influencing the risk of LTL and five major CVDs in the same or opposite directions and to explore whether locus-specific effects vary by racial/ethnic group in these regions of genetic overlap. Due to the smaller sample size, East Asian GWAS yielded fewer variants associated with LTL or CVDs compared to European GWAS. In East Asian individuals, FUMA further delineated 11 independent genomic risk loci as pleiotropic, spanning 7 unique chromosomal regions (Supplementary Data 26-27). Among the loci examined, 8 loci were linked to LTL and 4 loci to CVDs. Notably, a solitary locus showed overlap between LTL and CVDs, accounting for 12.50% and 25.00% of the total loci associated with each category. We found three promising loci shared across multiple trait pairs at 10q24.33, 3q26.2, and 1q42.12. For example, the pleiotropic locus at 10q24.33 (mapped gene: *SLK*) was jointly associated with LTL and all CVDs, excluding HF and PAD. The next promising locus is an intron of the leucine-rich repeat containing 34 (*LRRC34*) gene on 3q26.2 encompassing index variant rs9831661 ($P = 3.83\times10^{-11}$),

and this locus demonstrated associations with both LTL-CAD and LTL-stroke. This variant has been reported to act as a ribonuclease inhibitor in DNA repair, chromosomal stability, and heart development[5]. Most of the top SNPs (81.82%) affected two traits within the trait pair in opposite directions, demonstrating a widespread mixed pattern of allelic effect directions among the shared loci, which is consistent with insignificant genetic correlations. ANNOVAR category annotation across the 11 loci from East Asian ancestry revealed that most index SNPs were intergenic or intronic. Seven of 11 (63.64%) index SNPs were intergenic, and three (27.27%) were intronic. No index SNPs with CADD scores greater than 12 were identified, and rs79019201 on 10q24.33 had the highest CADD score (CADD score: 8.428). The four index SNPs had an RDB score of 7, indicating the least support for regulatory potential.

Further colocalization analysis showed that no pleiotropic locus had support (PP.H4 > 0.7), indicating that causal variants for these loci are not effectively tagged in East Asian populations. Two pleiotropic loci had PP.H3 greater than 0.7, suggesting that different causal variants are implicated in LTL and CVD.

We replicated the shared genetic pleiotropic loci observed in European ancestry individuals across trait pairs in East Asian ancestry individuals. Further, we discovered loci of genetic overlap exhibiting racial/ethnic differences. Out of the 11 shared loci identified in the East Asian ancestry, 9 were also identified in the European ancestry. Only two loci showed evidence of being East Asian-specific in this comparison, highlighting the importance of complementary ancestry-specific loci for identifying associations not shared across diverse populations. Additionally, five loci (three unique) initially identified in GWAS of AF, CAD, and stroke in European ancestry individuals have now been shown to be associated with East Asian populations as well.

Section 5. Pleiotropic genes associated with LTL and multiple CVDs of East Asian ancestry

We conducted MAGMA to analyze genes located within or overlapping the pleiotropic loci, using both PLACO outputs and single-trait GWAS to identify candidate pleiotropic genes. Our finding of significant gene-level pleiotropy identified 17 potential genes (10 unique), using 22 pleiotropic

genes located within or overlapping 11 pleiotropic loci. Among these, 12 genes were identified in two or more trait pairs (Supplementary Data 30-32). *COL17A1* and *SLK* were the top genes identified in three trait pairs, followed by *MYNN*, *LRRC34*, and *ACTRT3*, detected in two trait pairs. Notably, collagen type XVII alpha one chain (*COL17A1*) and STE20-like kinase (*SLK*) were located on the 10q24.33 locus, identified in all trait pairs except for LTL-HF and LTL-PAD. *SLK* (Ste20-like kinase), a serine/threonine protein kinase, has been identified as a novel kinase that phosphorylates RhoA, a significant regulator of cardiovascular functions. The activation of Rho proteins is a common element in the pathogenesis of hypertension[6]. In addition, through a novel signaling mechanism, *SLK*-mediated activation of the stress response pathway induces senescence by accumulating protein aggregates and causing oxidative and metabolic mitochondrial stress, DNA damage, and pro-inflammatory processes[7]. Modulating the integrated stress response is believed to slow aging and ameliorate age-related pathologies[8]. Of the pleiotropic genes identified, two (11.76%) were novel for LTL and 15 (88.24%) for CVDs. No pleiotropic gene had been previously reported as being associated with both traits. Furthermore, all genes identified by MAGMA were confirmed by FUMA positional mapping (Supplementary Data 28-29). The number of pleiotropic genes (n = 17) identified in East Asian ancestry was 96.44% less than in European ancestry (n = 478), possibly because the relatively small sample sizes of East Asian samples led to limited power for predicting genes. Most pleiotropic genes were ancestor-specific, especially, *SH2B3* was specific to Europeans but not included in the East Asians data set, highlighting population-specific disease genetic architecture; three genes (*COL17A1*, *SH3PXD2A*, *STN1*) were generally shared across ancestries.

Overall, both the SNP-level analysis and gene-level analysis in East Asian ancestry converged on the same relevant risk loci, the same potential pleiotropic variants, and the same risk genes.

Section 6. Shared biological pathways between LTL and five major CVDs of East Asian ancestry

Shared genetic determinants are also reflected in common biological pathways. Thus, gene set enrichment analysis was performed to identify potential biological pathways for the significant candidate genes identified by MAGMA. Gene-set analysis revealed that genes associated with

LTL and CAD were enriched in four Gene Ontology (GO) terms, including 'neutral lipid metabolic process,' 'renal system vasculature development,' 'glomerular mesangial cell proliferation,' and 'positive regulation of cardiac muscle cell differentiation,' with a predominance of gene-sets related to positive regulation of cardiomyocyte differentiation (Supplementary Data 33). Finally, no significant pathways were identified for AF, HF, PAD, and stroke. Results from MAGMA's gene set analysis using East Asian ancestry samples were compared with those from European ancestry. For most pathways, the enrichment patterns differ, suggesting potentially different genetic structures between European and East Asian populations.

The absence of a reference panel for East Asian ancestry in specific statistical software, including e-MAGMA, TWAS, and SMR, precluded the identification of tissue-specific genes and causal proteins. Beyond genetic signals shared across populations, our findings suggest the presence of racial/ethnic-specific differences in the pleiotropic effects influencing traits, providing new insights into the shared etiology of LTL and CVD in individuals from East Asian populations. However, we identified only a limited number of lead SNPs, genomic loci, overlapping genes, and gene sets between LTL and five CVDs in East Asian populations, which is expected given the small sample sizes and low power of the original GWAS. This limitation makes it challenging to interpret these shared genetic signals as indicative of a shared etiology of LTL and CVDs. Thus, there is an urgent need for expanded GWAS in the future to deepen our understanding of the genetic architecture underlying LTL and CVDs and putative shared etiological processes in East Asian populations, which could have significant implications for targeted therapies and clinical utility.

Supplementary References

1 Dorajoo, R. *et al.* Loci for human leukocyte telomere length in the Singaporean Chinese population and trans-ethnic genetic studies. Nat Commun 10, 2491, doi:10.1038/s41467-019-10443-2 (2019).

2 Ishigaki, K. *et al.* Large-scale genome-wide association study in a Japanese population identifies novel susceptibility loci across different diseases. *Nat Genet* **52**, 669-679, doi:10.1038/s41588-020-0640-3 (2020).

3 Low, S. K. *et al.* Identification of six new genetic loci associated with atrial fibrillation in the Japanese population. *Nat Genet* **49**, 953-958, doi:10.1038/ng.3842 (2017).

4 Zhang, Z. *et al.* Genome-wide association analyses identified novel susceptibility loci for pulmonary embolism among Han Chinese population. *BMC Med* **21**, 153, doi:10.1186/s12916-023-02844-4 (2023).

5 Paik, J. K., Kang, R., Cho, Y. & Shin, M. J. Association between Genetic Variations Affecting Mean Telomere Length and the Prevalence of Hypertension and Coronary Heart Disease in Koreans. *Clin Nutr Res* **5**, 249-260, doi:10.7762/cnr.2016.5.4.249 (2016).

6 Guilluy, C. *et al.* Ste20-related kinase SLK phosphorylates Ser188 of RhoA to induce vasodilation in response to angiotensin II Type 2 receptor activation. *Circ Res* **102**, 1265-1274, doi:10.1161/circresaha.107.164764 (2008).

7 Sabourin, L. A. & Rudnicki, M. A. Induction of apoptosis by SLK, a Ste20-related kinase. *Oncogene* **18**, 7566-7575, doi:10.1038/sj.onc.1203119 (1999).

8 Derisbourg, M. J., Hartman, M. D. & Denzel, M. S. Perspective: Modulating the integrated stress response to slow aging and ameliorate age-related pathology. *Nat Aging* **1**, 760-768, doi:10.1038/s43587-021-00112-9 (2021).
