# Supplementary material for: Contribution of leukocyte telomere length to cardiovascular disease onset from genome-wide cross-trait analysis
Source: Nat Commun. 2025 Sep 30;16:8677. doi: 10.1038/s41467-025-63707-5 (PMC12485159; doi:10.1038/s41467-025-63707-5)
Supplement: Supplementary file 2 — Description of Additional Supplementary Files [file 41467_2025_63707_MOESM2_ESM.pdf]

Description of Additional Supplementary Files

Supplementary Data 1. Overview of leukocyte telomere length and six cardiovascular diseases included in this study

Supplementary Data 2. Univariate and bivariate LDSC results for leukocyte telomere length and six cardiovascular diseases

Supplementary Data 3. Univariate and bivariate MiXeR results for leukocyte telomere length and six cardiovascular diseases

Supplementary Data 4. Univariate LAVA results for each leukocyte telomere length and six cardiovascular diseases

Supplementary Data 5. Bivariate LAVA results between leukocyte telomere length and six cardiovascular diseases

Supplementary Data 6. Causal relationship between leukocyte telomere length and six cardiovascular diseases

Supplementary Data 7. Genomic risk loci identified for PLACO results of leukocyte telomere length and six cardiovascular diseases by functional annotation of genomic risk loci using FUMA

Supplementary Data 8. Genomic risk loci identified for single-trait GWAS results of leukocyte telomere length and six cardiovascular diseases by functional annotation of genomic risk loci using FUMA

Supplementary Data 9. Prioritized genes identified for PLACO results of leukocyte telomere length and six cardiovascular diseases by mapping of genomic risk loci using

FUMA

Supplementary Data 10. Prioritized genes identified for single-trait GWAS results of leukocyte telomere length and six cardiovascular diseases by mapping of genomic risk loci using FUMA

Supplementary Data 11. Candidate pleiotropic genes identified for PLACO results of leukocyte telomere length and six cardiovascular diseases using MAGMA and e-MAGMA

Supplementary Data 12. Gene-based analysis for all genes that were nominally significant for PLACO results between leukocyte telomere length and six cardiovascular diseases

Supplementary Data 13. Gene-based analysis for all genes that were nominally significant for single-trait GWAS results of leukocyte telomere length and six cardiovascular diseases

Supplementary Data 14. S-LDSC-based heritability enrichment estimates in 49 tissue types or 489 functional categories for single-trait GWAS results of leukocyte telomere length and six cardiovascular diseases using LDSC-SEG

Supplementary Data 15. e-MAGMA analysis for 10 related tissue-specific genes that were nominally significant for PLACO results between telomere length and six cardiovascular diseases

Supplementary Data 16. Transcriptome-wide association study analysis for 10 related tissue-specific genes that were nominally significant for single-trait GWAS results of leukocyte telomere length and six cardiovascular diseases

Supplementary Data 17. Biological processes and enriched signaling pathways between leukocyte telomere length and six cardiovascular diseases

Supplementary Data 18. Proteome-wide MR analysis for causalities of plasma protein levels with leukocyte telomere length and six cardiovascular diseases including sensitivity analysis

Supplementary Data 19. HyPrColoc results of leukocyte telomere length and six cardiovascular diseases

Supplementary Data 20. Overview of leukocyte telomere length and five cardiovascular diseases in East Asian populations included in this study

Supplementary Data 21. Univariate and bivariate LDSC results for leukocyte telomere length and five cardiovascular diseases in East Asian populations

Supplementary Data 22. Univariate and bivariate MiXeR results for leukocyte telomere length and five cardiovascular diseases in East Asian populations

Supplementary Data 23. Univariate LAVA results for each of leukocyte telomere length and five cardiovascular diseases in East Asian populations

Supplementary Data 24. Bivariate LAVA results between leukocyte telomere length and five cardiovascular diseases in East Asian populations

Supplementary Data 25. Causal relationship between leukocyte telomere length and five cardiovascular diseases in East Asian populations

Supplementary Data 26. Genomic risk loci identified for PLACO results of leukocyte telomere length and five cardiovascular diseases in East Asian populations by

functional annotation of genomic risk loci using FUMA

Supplementary Data 27. Genomic risk loci identified for single-trait GWAS results of leukocyte telomere length and five cardiovascular diseases in East Asian populations by functional annotation of genomic risk loci using FUMA

Supplementary Data 28. Prioritized genes identified for PLACO results of leukocyte telomere length and five cardiovascular diseases in East Asian populations by mapping of genomic risk loci using FUMA

Supplementary Data 29. Prioritized genes identified for single-trait GWAS results of leukocyte telomere length and five cardiovascular diseases in East Asian populations by functional annotation and mapping of genomic risk loci using FUMA

Supplementary Data 30. Candidate pleiotropic genes identified for PLACO results of leukocyte telomere length and five cardiovascular diseases in East Asian populations using MAGMA

Supplementary Data 31. Gene-based analysis for all genes that were nominally significant for PLACO results between leukocyte telomere length and five cardiovascular diseases in East Asian populations

Supplementary Data 32. Gene-based analysis for all genes that were nominally significant for single-trait GWAS results of leukocyte telomere length and five cardiovascular diseases in East Asian populations

Supplementary Data 33. Gene sets significantly associated with PLACO results between leukocyte telomere length and five cardiovascular diseases in East Asian populations based on the susceptibility genes using MAGMA
