# Supplementary material for: Contribution of leukocyte telomere length to cardiovascular disease onset from genome-wide cross-trait analysis
Source: Nat Commun. 2025 Sep 30;16:8677. doi: 10.1038/s41467-025-63707-5 (PMC12485159; doi:10.1038/s41467-025-63707-5)
Supplement: Supplementary file 36 — Reporting Summary [file 41467_2025_63707_MOESM36_ESM.pdf]

nature portfolio

Corresponding author(s): Siim Pauklin, Yuliang Feng

Last updated by author(s): Jul 27, 2025

Reporting Summary

Nature Portfolio wishes to improve the reproducibility of the work that we publish. This form provides structure for consistency and transparency in reporting. For further information on Nature Portfolio policies, see our Editorial Policies and the Editorial Policy Checklist.

Statistics

For all statistical analyses, confirm that the following items are present in the figure legend, table legend, main text, or Methods section.

n/a	Confirmed	
☐	☒	The exact sample size (*n*) for each experimental group/condition, given as a discrete number and unit of measurement
☒	☐	A statement on whether measurements were taken from distinct samples or whether the same sample was measured repeatedly
☐	☒	The statistical test(s) used AND whether they are one- or two-sided<br>*Only common tests should be described solely by name; describe more complex techniques in the Methods section.*
☒	☐	A description of all covariates tested
☐	☒	A description of any assumptions or corrections, such as tests of normality and adjustment for multiple comparisons
☐	☒	A full description of the statistical parameters including central tendency (e.g. means) or other basic estimates (e.g. regression coefficient) AND variation (e.g. standard deviation) or associated estimates of uncertainty (e.g. confidence intervals)
☐	☒	For null hypothesis testing, the test statistic (e.g. *F*, *t*, *r*) with confidence intervals, effect sizes, degrees of freedom and *P* value noted<br>*Give P values as exact values whenever suitable.*
☒	☐	For Bayesian analysis, information on the choice of priors and Markov chain Monte Carlo settings
☒	☐	For hierarchical and complex designs, identification of the appropriate level for tests and full reporting of outcomes
☒	☐	Estimates of effect sizes (e.g. Cohen's *d*, Pearson's *r*), indicating how they were calculated

Our web collection on statistics for biologists contains articles on many of the points above.

Software and code

Policy information about availability of computer code

Data collection	No software was used for data collection.
Data analysis	All software (and version, where applicable) used to conduct the analyses in this paper are freely available online: LDSC (v1.0.1; https://github.com/bulik/ldsc), MiXeR (v1.3; https://github.com/precimed/mixer), LAVA (v0.1.0; https://github.com/josefin-werme/LAVA), LCV (https://github.com/lukejoconnor/LCV), MRlap (v0.0.3; https://github.com/n-mounier/MRlap), TwoSampleMR (v0.6.8; https://mrcieu.github.io/TwoSampleMR), PLACO (v0.1.1; https://github.com/RayDebashree/PLACO), FUMA (v1.5.4; http://fuma.ctglab.nl/), HyPrColoc(v1.0; https://github.com/jrs95/hyprcoloc), MAGMA (v.1.08; https://ctg.cncr.nl/software/magma), e-MAGMA (https://github.com/eskederks/eMAGMA-tutorial), TWAS (http://gusevlab.org/projects/fusion/), SMR (v1.31; https://yanglab.westlake.edu.cn/software/smr/), COLOC (v5.2.1; https://github.com/chr1swallace/coloc), and R (v.4.1.3; https://www.r-project.org/).

For manuscripts utilizing custom algorithms or software that are central to the research but not yet described in published literature, software must be made available to editors and reviewers. We strongly encourage code deposition in a community repository (e.g. GitHub). See the Nature Portfolio guidelines for submitting code & software for further information.

Data

Policy information about availability of data

All manuscripts must include a data availability statement. This statement should provide the following information, where applicable:

- Accession codes, unique identifiers, or web links for publicly available datasets
- A description of any restrictions on data availability
- For clinical datasets or third party data, please ensure that the statement adheres to our policy

All analyses in this study were conducted using publicly available datasets. Download links for publicly available GWAS summary statistics used as inputs in this study are provided in Supplementary Data 1. The study used only openly available GWAS summary statistics on leukocyte telomere length and six major cardiovascular diseases that have originally been conducted using human data. GWAS summary statistics on LTL are available at https://figshare.com/s/caa99dc0f76d62990195. Genome-wide summary statistics for AF, HF, and stroke are available from the GWAS Catalog under accession codes GCST006414, GCST009541, and GCST90104539, respectively. GWAS summary statistics for CAD and PAD are publicly available for download from the Cardiovascular Disease Knowledge Portal (CVDKP) at https://cvd.hugeamp.org/datasets.html. Genome-wide summary statistics for VTE were obtained from the deCODE Genetics website: https://www.decode.com/summarydata/. Blood-based cis-pQTL from UKB-PPP are obtained from https://www.synapse.org/Synapse:syn51365303. Source data used for generating the figures are available in the Source Data file with this paper.

Research involving human participants, their data, or biological material

Policy information about studies with human participants or human data. See also policy information about sex, gender (identity/presentation), and sexual orientation and race, ethnicity and racism.

Reporting on sex and gender	We did not perform sex- or gender-stratified analyses; instead, all individuals were analyzed as a single combined population.
Reporting on race, ethnicity, or other socially relevant groupings	The GWAS datasets used in our analyses are predominantly of European ancestry.
Population characteristics	For our primary analysis, we utilized published GWAS datasets derived from large population- or cohort-based studies. Detailed information on these datasets is provided in Supplementary Data 1.
Recruitment	The GWAS summary statistics used in our study were obtained from previously published datasets; no new participant recruitment was conducted as part of the current study.
Ethics oversight	The research reported herein was conducted in compliance with all applicable ethical standards.

Note that full information on the approval of the study protocol must also be provided in the manuscript.

Field-specific reporting

Please select the one below that is the best fit for your research. If you are not sure, read the appropriate sections before making your selection.

☐ Life sciences ☒ Behavioural & social sciences ☐ Ecological, evolutionary & environmental sciences

For a reference copy of the document with all sections, see nature.com/documents/nr-reporting-summary-flat.pdf

Behavioural & social sciences study design

All studies must disclose on these points even when the disclosure is negative.

Study description	The study adopted a quantitative design, utilizing statistical analysis of genome-wide association study (GWAS) summary statistics across leukocyte telomere length (LTL) and six cardiovascular diseases (CVDs). Our study aimed to explore whether LTL and CVDs share a common genetic architecture, and to disentangle the contributions of vertical (causal relationship) and horizontal (shared genes and biological pathway) pleiotropy. Specifically, we asked whether the genetic variants associated with LTL are also implicated in CVD risk, and if so, through which pleiotropic mechanisms.
Research sample	All European participants with the LTL measured from a UKB baseline sample were included. GWAS summary statistics on LTL are available at https://figshare.com/s/caa99dc0f76d62990195. Data consists of case-control GWAS summary statistics of six major cardiovascular diseases of European ancestry. We selected GWAS datasets based on mainly two considerations: the highest sample size and the most stringent diagnosis definition criteria. Specifically, genome-wide summary statistics for AF, HF, and stroke are available from the GWAS Catalog under accession codes GCST006414, GCST009541, and GCST90104539, respectively. GWAS summary statistics for CAD and PAD are publicly available for download from the Cardiovascular Disease Knowledge Portal (CVDKP) at https://cvd.hugeamp.org/datasets.html. Genome-wide summary statistics for VTE were obtained from the deCODE Genetics website: https://www.decode.com/summarydata/.
Sampling strategy	Not applicable as we used previously collected GWAS data. We selected GWAS datasets based on mainly two considerations: the highest sample size and the most stringent diagnosis definition criteria.

Data collection	Not applicable as we used previously collected GWAS data. Case status was defined through diagnostic assessment by a clinician and self-reports. For information on the individual traits, we refer to the primary publications listed in Supplementary Data 1.
Timing	Not applicable as we used previously collected GWAS data. Our screening of available GWAS summary statistics ended in Jan. 2024.
Data exclusions	We selected GWAS datasets based on mainly two considerations: the highest sample size and the most stringent diagnosis definition criteria.
Non-participation	No participants were directly involved in this study.
Randomization	Not applicable as we used previously collected GWAS data, which we were not involved in the collection of. Original GWAS study designs are not randomized, as participants are deemed case or control based on their respective diagnosis status.

Reporting for specific materials, systems and methods

We require information from authors about some types of materials, experimental systems and methods used in many studies. Here, indicate whether each material, system or method listed is relevant to your study. If you are not sure if a list item applies to your research, read the appropriate section before selecting a response.

Materials & experimental systems

n/a	Involved in the study
☒	☐ Antibodies
☒	☐ Eukaryotic cell lines
☒	☐ Palaeontology and archaeology
☒	☐ Animals and other organisms
☒	☐ Clinical data
☒	☐ Dual use research of concern
☒	☐ Plants

Methods

n/a	Involved in the study
☒	☐ ChIP-seq
☒	☐ Flow cytometry
☒	☐ MRI-based neuroimaging

Plants

Seed stocks	n/a
Novel plant genotypes	n/a
Authentication	n/a
